# Wild‐type p53 regulates OTOP2 transcription through DNA loop alteration of the promoter in colorectal cancer

**DOI:** 10.1002/2211-5463.12554

**Published:** 2018-12-20

**Authors:** Huajun Qu, Yi Su, Lianzhi Yu, Hongchao Zhao, Chunxia Xin

**Affiliations:** ^1^ Department of Oncology Yuhuangding Hospital of Yantai Shandong China; ^2^ Department of Radiotherapy Yuhuangding Hospital of Yantai Shandong China; ^3^ Department of Physical Examination Yuhuangding Hospital of Yantai Shandong China; ^4^ Department of Gastroenterology The First Affiliated Hospital of Zhengzhou University Henan China

**Keywords:** chromatin, colorectal cancer, DNA loop, OTOP2, p53

## Abstract

Colorectal cancer (CRC) is the third most commonly diagnosed malignancy worldwide and remains a major public health issue. Therefore, further investigation is required to delineate the cellular and molecular mechanisms underlying colorectal tumorigenesis. Using CRC data taken from The Cancer Genome Atlas, we determined that the expression of otopetrin 2 (OTOP2) is highly correlated with malignancy grade and rate of patient survival. Here, we report that OTOP2 is down‐regulated in cancerous tissues and that elevated OTOP2 effectively suppresses tumor proliferation *in vitro*. We demonstrate that wild‐type p53 (wtp53), but not mutant p53 (mtp53), can regulate the transcription of *otop2* in CRC cells. Subsequently, we investigate the chromatin architecture of the *otop2* promoter, whereby we discover alterations in p53‐dependent DNA loop organization and CCCTC‐binding factor (CTCF) binding between cells with wtp53 and mtp53. In conclusion, our study promotes an in‐depth understanding of tumorigenesis, which may also lead to the development of therapeutic applications targeting human malignancy.

**Abbreviations**
3C: chromatin conformation capture
CBS: consensus binding sites
ChIP: chromatin immunoprecipitation
CRC: colorectal cancer
CTCF: CCCTC‐binding factor
EdU: 5‐ethynyl‐2′‐deoxyuridine
mtp53: mutated p53
OTOP2: otopetrin 2
TCGA: The Cancer Genome Atlas
wtp53: wild‐type p53

Colorectal cancer (CRC) is currently the third most commonly diagnosed cancer worldwide 1 and leads to approximately 700 000 deaths and 1.4 million newly diagnosed cases each year 2. With rapid technological advancements in next‐generation sequencing, numerous omics‐related fields have contributed to important discoveries in cancer biology. Data taken from The Cancer Genome Atlas (TCGA) illustrate the multiple dimensions portrayed by tumorigenesis and assist in the identification of tumorigenic biomarkers by analyzing their differential expression within tumor, paracarcinoma, and normal tissue samples.

p53, one of the most widely studied transcription factors, has been shown to regulate the transcription of multiple genes to orchestrate responses to stress. This is achieved through binding at specific regions, which can regulate epigenetic modifications. Conversely, aberrant gain of function identified in mutant p53 (mtp53) has been shown to promote tumorigenesis; however, many of its downstream effects are still unknown 3. Previous investigation has revealed that the transcription of p53 targets is regulated by the insulator protein CCCTC‐binding factor (CTCF) and the cohesion complex‐mediated chromatin boundary signature at the p53 binding domain 4. Despite this, further study is required to understand the importance of spatial organization changes to the p53 binding domains throughout disease progression, especially those at distributed sites within regulatory elements.

Here, we demonstrated that otopetrin 2 (*otop2*) plays an important role in CRC development. In this study, we validated that *otop2* is remarkably down‐regulated and investigated a series of p53 cognitive DNA motifs using chromatin conformation capture (3C) to analyzed DNA loop organization. Furthermore, we observed the impact of impaired p53 activity on chromatin organization in CRC cell lines. Taken together, our results suggest that p53 influences transcriptional regulation via the alteration of chromatin architecture. Therefore, we aimed to elucidate the relationships between *otop2* and p53 to provide a better understanding of the role played by this novel p53 target gene in CRC.

## Materials and methods

### Cell lines, RNA oligos, antibodies, chemicals, and vectors

The CRC cell lines DLD1, LS174T, SW403, and SW480 were obtained from American Type Culture Collection (ATCC, Manassas, VA, USA). *otop2* siRNA 5′‐GCGGATGCCCTTCGTACATTA‐3′ was obtained (InvivoGen, San Diego, CA, USA). OTOP2, PCNA, p21, and GAPDH primary antibodies were purchased from NOVUS Biologicals (Littleton, CO, USA), and ChIP grade antibodies for p53, OTOP2, CTCF, and RNA pol II were purchased from Abcam (Cambridge, UK). Secondary antibodies for WB and IF were all purchased from Roche (Basel, Switzerland). Crystal violet powder and mounting medium with DAPI solution were both obtained from SCR Co., Ltd (Sinopharm Chemical Reagent, Shanghai, China). Endonuclease of XmaI, T4 DNA ligase, and High Capacity cDNA Reverse Transcription Kit were purchased from Takara (Kusatsu, Japan). PCR master mix with SYBRGreen, 96‐well plates, RPMI‐1640, Lipofectamine 3000, protein A beads, Dulbecco's modified Eagle's medium (DMEM), and FBS were purchased from Thermo Fisher Scientific (Waltham, MA, USA). The enhanced chemiluminescence detection kit was obtained from Millipore (Burlington, VT, USA). pGL3 luciferase reporter system was purchased from Promega (Madison, WI, USA). And the pcDNA 3.1 vector for *otop2* overexpression was purchased from Thermo Fisher Scientific. For plasmids construction, full‐length *otop2* and p53 transcripts were cloned from DLD1 cells and linked into pcDNA3.1, respectively, using the Gibson Assembly Cloning Kit (NEB, Ipswich, MA, USA). Primers for mutagenesis were used to generate the mutations of R273H and P309S based on wtp53 via PCR under the following condition; 95 °C 5 min for initial denaturation, followed by 35 cycles of 95 °C for 30 s, 58 °C for 1 min, and 72 °C for 6 min. Subsequently, the DNA mixture and/or PCR products were transformed into competent cells and the clones were picked up from ampicillin‐positive agar plates for identification.

### Bioinformatic study

mRNA profiles and clinical data of 274 colon cancer and 41 normal samples from TCGA were analyzed for comparison and correlation analysis using c‐BioPortal Program 5, 6. The heatmap was constructed with HemI 1.0 tools (http://hemi.biocuckoo.org/).

### Cell culture and transfection

DLD1 cells were cultured in RPMI‐1640, and LS174T, SW403, and SW480 cells were cultured in DMEM with 10% FBS in 37 °C with 5% CO_2_. pcDNA3.1 with the *otop2* coding sequence or p53 as well as pGL3 with p53 consensus binding sites (CBS) was cloned and transfected accordingly using Lipofectamine 3000 and harvested following a 48‐h transfection period.

### Reporter imaging assays

The full‐length 2.1‐kb sequence upstream of the OTOP2 promoter (chr16: 50546330 to 50548429, GRCh38) and three p53 binding sites within this promoter region (CBS1: −1322 to −1180, CBS2: −3100 to −2740, and CBS3: −3620 to −3360 by considering TSS as 0) were cloned into the pGL3 promoter–reporter (Promega), respectively, and mixed with either pcDNA3‐wtp53 or mtp53 (R273H) and (P309S), as well as pGL3 (Renilla luciferase‐expressing construct; Promega), then cotransfected into SW403 p53 null cells. Following a 48‐h transfection period, the Renilla luciferase substrate coelenterazine (1.5 μg·mL^−1^) was added to examine the luciferase activity as a measure of transfection efficiency. D‐luciferin (100 μg·mL^−1^) was used for the detection of p53‐driven reporter activation, and bioluminescent images were captured using the Xenogen IVIS system.

### Crystal violet staining

Cells were fixed in 3.7% paraformaldehyde at room temperature (RT) for 10 min and stained for 30 min using 0.05% crystal violet solution. Following this, cells were washed and air‐dried. The dishes were photographed to estimate the colony count.

### 5‐Ethynyl‐2′‐deoxyuridine (EdU) assay

Ten millimolar stock solution of Edu was prepared in DMSO. The final concentration of 10 μm EdU was diluted by methanol, and 5 μm Hoechst 33342 was added in the cultured cells for 3 h. Following incubation, cells were fixed in 3.7% paraformaldehyde at RT for 10 min and permeabilized using 0.5% Triton X‐100 then washed with PBS. The cells were observed under an Olympus BX‐51 microscope (Olympus, Tokyo, Japan). The excitation wavelength of EdU and Hoechst was 594 and 350 nm. Images were acquired and analyzed using ImageJ software. The level of EdU‐positive staining was normalized by Hoechst.

### Immunofluorescence assay

Cells were passaged and grown in six‐well dishes containing a sterilized cover glass. Following a 48‐h transfection, the glasses were gently washed with PBS and fixed with 2% paraformaldehyde at RT for 10 min and permeabilized with 0.1% Triton X‐100. Cells were blocked in 2% horse serum PBS for 30 min and subsequently incubated with OTOP2 antibody overnight at 4 °C. Following this, cells were washed and incubated with the appropriate Alexa Fluor secondary antibody at 1 : 10 000 dilutions for 30 min in RT. Cells were washed again and mounted in mounting medium with DAPI. Cells were observed under an Olympus BX‐51 microscope, and images were acquired and analyzed using imagej software.

### RNA extraction

Total RNA was extracted using Trizol and quantified using the Nanodrop. One hundred nanogram RNA was used to conduct reverse transcription by High Capacity cDNA Reverse Transcription Kit. One microlitre of products was used as the template for PCR preparation. Primers are listed in Table 1.

**Table 1:** All the primers used in this study are listed in this table

Purpose	Primer	Sequence	Tm (°C)
For cloning	p53 CDS	ATGGAGGAGCCGCAGTCAGAT	55
		TCAGTCTGAGTCAGGCCCTT	
	p53_ R273H	ACGGAACAGCTTTGAGGTGCATGTTTGTGCCTGTCCTGGG	62
		CCCAGGACAGGCACAAACATGCACCTCAAAGCTGTTCCGT	
	p53_P309S	CACTAAGCGAGCACTGTCCAACAACACCAGCTCCTC	64
		GAGGAGCTGGTGTTGTTGGACAGTGCTCGCTTAGTG	
	OTOP2 CDS	ATGTCCGAGGAGCTGGCCCA	52
		TCAGGACAGCACGTAGACCT	
	CBS1 (−1322 to −1180)	AAAGGTCGGGGCTGGTCTC	55
		GCTCCCCGCTGCAGTTCGCCA	
	CBS2 (−3100 to −2740)	CAAATAAGAAACTAGAGGAGTG	58
		CGTATTGGGACAGAACAGCCC	
	CBS3 (−3620 to −3360)	GTCGCCAGCCCTGGAGCTCGG	56
		GCATCAAAGGCAAGGCTGGTTG	
	OTOP2 promoter full length	CTTTGTGACCTTGGGCAAGTG	56
		GACGCGCTCCTGCCGCCGTCGCCG	
For mRNA detection	p53_detection	CCTGCCCTGT GCAGCTGTGG G	60
		CCCACAGCTGCACAGGGCAGG	
	OTOP2_detection	AGTCAGCCATCAAGATCCTG	60
		CCACATTCTTCCACATGACATA	
	Gapdh	CGGAGTCAACGGATTTGGTCGTAT	60
		AGCCTTCTCCATGGTGGTGAAGAC	
For ChIP assay	CTCF_1 (−2832 to −2688)	AAGACTCCTCCGGAGCTCTC	58
		GGAGCTGTTGAGACAAGGGA	
	CTCF_2 (−2698 to −2520)	TCCCTTGTCTCAACAGCTCC	55
		TGAGGCCAAGGCTGGCT	
	CTCF_3 (−1644 to −1455)	GGAACCCCCAAATCCCG	56
		TTAGACCCTGGCGTTT	
	CTCF_4 (−1122 to −900)	CCGCCAGCCCCCGCTGC	56
		GGCGGCTCCCTGGAC	
	CTCF_5 (−884 to −720)	GCCGCGCACTGTGACGCCC	55
		GGCACCCCTCCCCAGCTCTC	
	CTCF_6 (−698 to −520)	CCGACCGCGGTTCGGTC	55
		ACGCGGAGCCGCTGCCAGTC	
	CTCF_7 (−498 to −251)	ACTGGGGTTGCCATCCCA	56
		GAGCCCCGAGAGCTAAGCTC	
	RNA pol II (−156 to −31)	AGCCAAACTCCCGCAGCT	56
		CTCCCACGCCGAGATGGACAGG	
	Negative control for ChIP assay	AGCTTCATCGGGATC	56
		TGAGGGTACAACTGA	
For 3C assay	3C_1	TGGGTCTCTAAGCCCAGAGAGTG	55
		CACTCTCTGGGCTTAGAGACCCA	
	3C_2	CCAGGCCCTGAACGGAGTC	56
		GACTCCGTTCAGGGCCTGG	
	3C_3	GTGCTCCGCCTTTGGATC	56
		GATCCAAAGGCGGAGCAC	
	Beta‐globin as the Positive control for 3C assay	ATTGTCTGATCTGATCTA	58
		TGACCCGCCATCCTGA	
	Negative control for 3C assay	ACTGACTTTAAAGCGGGTAC	60
		GTACCCGCTTTAAAGTCAGT	

The gene location is based on TSS as 0.

### Chromatin immunoprecipitation (ChIP) assay

The ChIP assay was performed as described 7. In brief, 10% whole cell lysates were saved as input after genomic DNA was broken into 200–500 bp by sonication. One microgram of antibodies was incubated with the rest of the lysate overnight, followed by 2‐h protein A beads incubation at 4 °C for target protein pull down. Primers were designed to encompass ~ 150 bp around the target regions of *otop2* promoter. Their sequences are listed in Table 1.

### Chromosome conformation capture (3C) assay

3C methods were operated as previously described 8. Cells were fixed and permeabilized, followed by an immunofluorescence assay, and then treated with 100 U XmaI (*otop2* promoter region (upstream 4000 bp from TSS) having four XmaI cutting sites) at 37 °C with slow rotation for 4 h and blocked by SDS. Following this, we conducted ligation for an additional 4 h to produce an interaction *in situ*. DNA was purified with ethanol and sonicated into fragments less than 500 bp, and detected by specific 3C primers (Table 1).

### Real‐time PCR

Analysis of DNA templates taken from RNA reverse transcription, and pull‐down samples of ChIP and 3C were conducted via qPCR using the QuantStudio 3 system (Thermo Fisher Scientific). In accordance with the given instructions, 95 °C for 30 s for initial denaturation, followed by 40 cycles at 95 °C for 5 s, appropriate annealing temperatures (Table 1) of 10 s and 72 °C, then 30 s were setup for PCR conditions. *C*_t_ values were harvested and calculated by using the delta–delta method. The expression of *gapdh* was used as a quality control.

### Western blotting

Cells at 70–80% confluence were washed with cold PBS and directly harvested using 10% SDS with bromophenol blue. Cell lysates were transferred into 1.5‐mL tubes for storage at −20 °C. Protein samples were separated by SDS/PAGE and transferred to polyvinylidene fluoride membranes. Following this, membranes were blocked in 5% nonfat milk TBS and then incubated with the respective primary antibodies (1 : 2000) overnight at 4 °C. Subsequently, membranes were washed with TBST and incubated with secondary antibodies (1 : 10 000) for 1 h at RT. Membranes were then treated with an enhanced chemiluminescence detection kit, and the intensity of each band was quantified by densitometry. Relative expression was normalized to that of GAPDH (1 : 10 000).

### Statistical analysis

All statistical analysis was conducted in spss 20 (IBM, Armonk, NY, USA). For real‐time PCR data, the 2^∆∆^ method was used to calculate the expression, enrichment, and probable DNA contact. Samples were analyzed using a one‐way ANOVA to evaluate the difference between groups. *P*‐value less than 0.05 was considered significant.

## Results

### Otop2 expression is closely related to CRC

**Figure 1:**
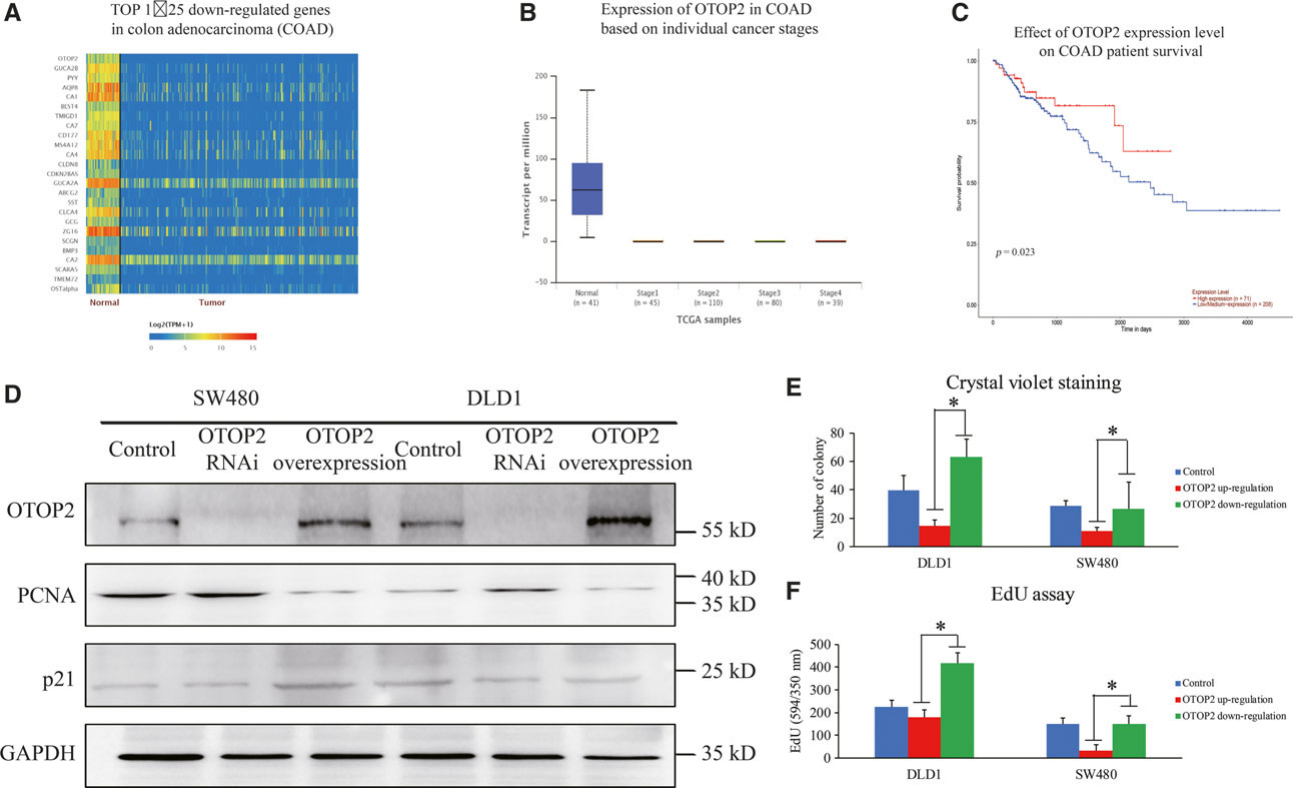
The close relationship between *otop2* and colon cancer. The heatmap of *otop2* mRNA profiles in normal and colon cancer tissues from TCGA database (A). The expression of *otop2* among the normal control and different stages of colon cancers (B). The mRNA levels of OTOP2 in colon cancer patients were separated into two groups with low and high expression, respectively. The survival period between the two groups was compared to evaluate the effect of *otop2* in colon cancer development (C). The influence of cell proliferation upon OTOP2 overexpression in CRC cell lines exhibited by WB (D) and crystal violet staining (E). Positive crystal violet staining samples were quantified and analyzed (F). All the data are presented as mean ± SEM of triplicate individual experiments. “*” represents *p* value less than 0.05 by one‐way ANOVA.

Initially, we analyzed 274 cases of colon adenocarcinoma from the TCGA database and found that the expression of *otop2* was significantly reduced in CRC tissues compared to normal control (Fig. 1A,B). In addition, we established that there was a significant negative correlation with the survival of colon cancer patients and *otop2* expression (Fig. 1C). Next, we investigated the role of overexpression of *otop2* and inhibition of OTOP2 expression in DLD1 and SW480 cells. We found that endogenous OTOP2 was extremely low in SW480 cells compared to DLD1 cells. Furthermore, we also observed reduced expression of PCNA in both SW480 and DLD1 cells as well as enhanced p21 protein expression in only SW480 cells following OTOP2 up‐regulation and vice versa (Fig. 1D). Consistently, the inhibitory effect of OTOP2 on cell proliferation was also observed using crystal violet staining and an EdU assay (Fig. 1E,F). Taken together, the results above indicate that *otop2* may act as a tumor suppressor.

### The transcription of otop2 is regulated by wild‐type p53

**Figure 2:**
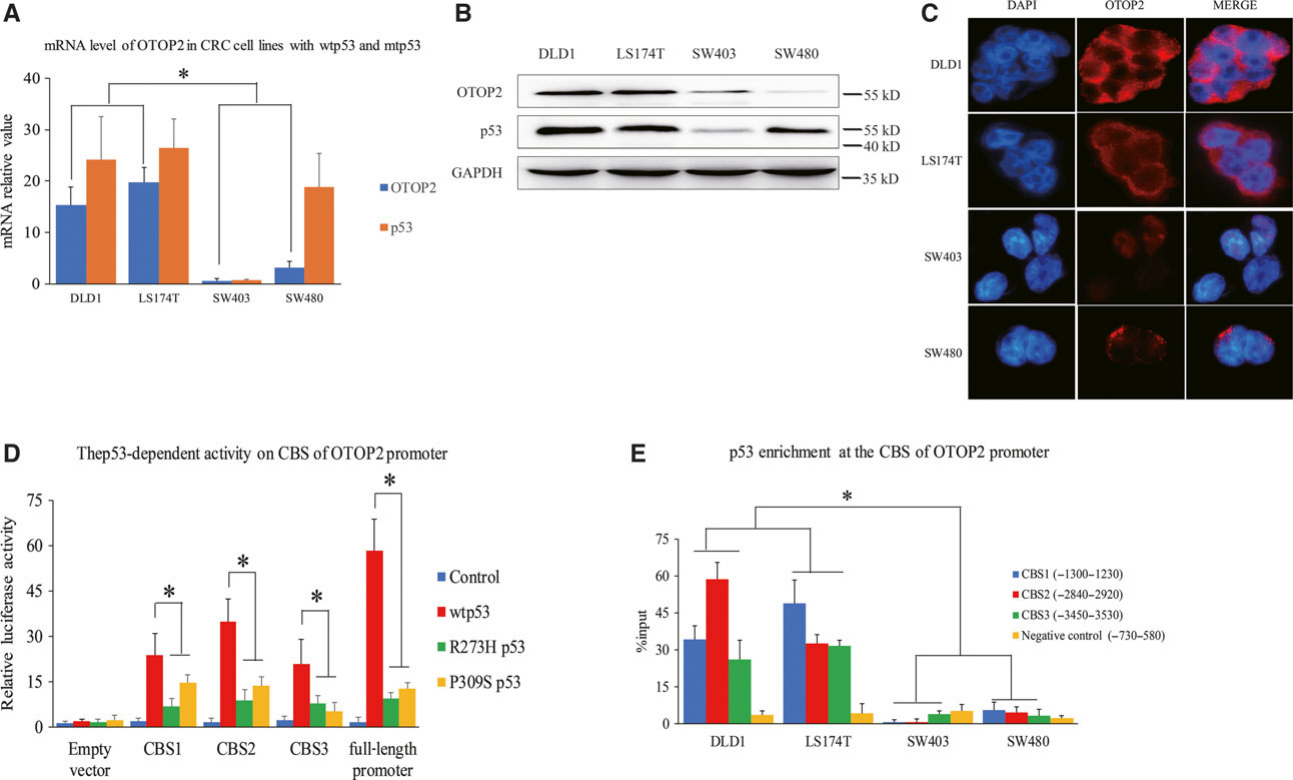
p53‐mediated *otop2* transcriptional regulation. The mRNA and protein expression of p53 and OTOP2 in CRC cell lines expressing either wtp53 or mtp53 using real‐time PCR (A), WB (B) and IF assay with 200× amplification (C). Three putative p53 consensus binding sequences of the *otop2* promoter (CBS1‐3) were inserted upstream of the minimal SV40 promoter in the pGL‐3 luciferase reporter plasmid cotransfected with wtp53 or mtp53 (R273H and P309S), respectively, in SW403 (p53 null) cells. The p53‐dependent activity on CBS at the *otop2* promoter region demonstrated using an imaging reporter assay (D). The binding status between endogenous p53 and the CBS1‐3 of the *otop2* promoter in wtp53 and mtp53 CRC cells using ChIP assay (E). All the data are presented as mean ± SEM of triplicate individual experiments. “*” represents *p* value less than 0.05 by one‐way ANOVA.

We acquired the promoter sequence of *otop2* and evaluated its underlying regulatory mechanism using ALGGEN (http://alggen.lsi.upc.es/). Previously, we analyzed potential transcription factors including p53 and noticed an extremely low expression of endogenous OTOP2 in DLD1 cells with wild‐type p53 (wtp53), compared to SW480 cells with mtp53 of R273H and P309S (Fig. 1D). Following this, CRC cell lines with either wtp53 or mtp53 were analyzed by sequencing to confirm our hypothesis of the potential relationship between OTOP2 and p53. Similarly, in this study we observed that OTOP2 was more highly expressed in DLD1 and LS174T CRC cell lines (wtp53) compared to SW403 (p53 null) and SW480 CRC cells (mtp53) (Fig. 2A–C). Therefore, we aimed to investigate the potential role of wtp53 in the transcriptional regulation of *otop2*. The predicted p53 binding domains within the *otop2* promoter were found to contain three putative consensus binding sequences (CBS1: −1250 ± 15, CBS2: −2900 ± 15, and CBS3: −3500 ± 15 by considering TSS as 0). These were cloned individually and inserted into the upstream minimal SV40 promoter using a pGL‐3 luciferase reporter plasmid. Interestingly, all three CBS were significantly activated by wtp53 but not by R273H and P309S (these two mutations were acknowledged in SW480 cells), or SW403 cells transfected with mtp53 (Fig. 2D). To further characterize this interaction, ChIP assay experiments were conducted to investigate the enrichment of p53 on the CBS. Similarly, the interaction between the CBS and p53 was highly enriched in DLD1 and LS174T cells compared to SW403 and SW480 cells (Fig. 2E). Collectively, we suggest that wtp53 enhances *otop2* expression via promoter interaction.

### p53‐mediated DNA loop alteration impacts otop2 promoter architecture modulated by CTCF in CRC cells

**Figure 3:**
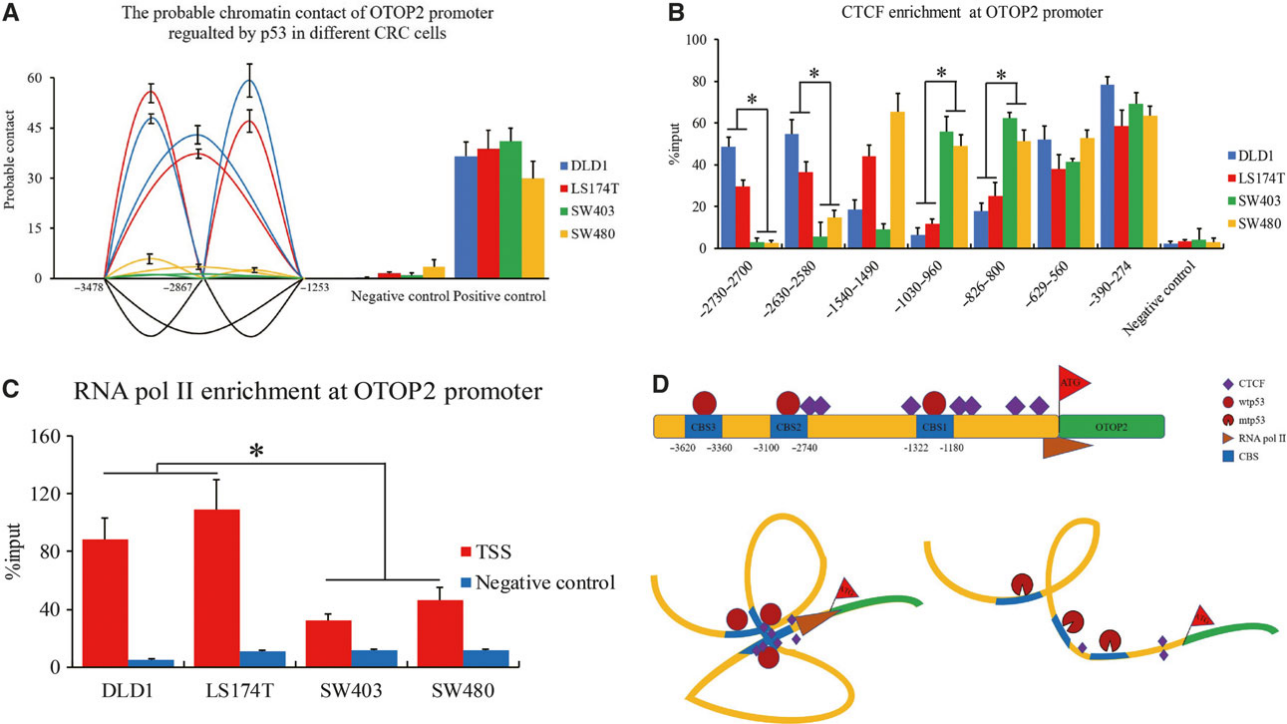
p53‐mediated DNA loop organization of the *otop2* promoter. DNA probable contact on the p53 CBS of the *otop2* promoter demonstrated by a 3C assay (A). CTCF (B) and RNA pol II (C) enrichment at the *otop2* promoter region using ChIP assay. The scheme of the p53‐mediated *otop2* promoter loop organization. Top: the overview of the binding sites of CTCF and p53 at the *otop2* promoter region. Bottom left: the CTCF‐mediated DNA loop organization recruited by wtp53. Bottom right: represents failure of the chromatin structure to form appropriately due to mtp53 (D). All the data are presented as mean ± SEM of triplicate individual experiments. “*” represents *p* value less than 0.05 by one‐way ANOVA.

Since the three CBS of p53 in the *otop2* promoter were identified, we raised the question whether p53 might recruit these binding domains together and consequently establish a distal proximity at the *otop2* promoter. Hence, we performed the 3C assay to investigate this p53‐mediated chromatin architecture. We observed that a substantial DNA–DNA proximity of the three CBS occurred in DLD1 and LS174T but not in SW403 and SW480 cells (Fig. 3A). This suggests that p53 binding is capable to modulate the chromatin conformation of *otop2*. Furthermore, we selected a series of CTCF binding sites located at the *otop2* promoter from all known CTCF ChIP‐seq (CTCFBSDB database, http://insulatordb.uthsc.edu/). Following this, we investigated CTCF and RNA pol II enrichment at the *otop2* promoter region using ChIP assay. We observed that CTCF binding status changed between wtp53 and mtp53 CRC cells (Fig. 3B). Additionally, we observed higher enrichment of CTCF at the CTCF binding sites near −2700 and −2600 while inverse alteration at −1000 and −800 of the *otop2* promoter in DLD1 and LS174T cells compared with SW403 and SW480 cells. Interestingly, we found that the CTCF binding status was p53 independent at −600 and −300 as well as cell type specific at −1500 of the *otop2* promoter. Finally, RNA pol II enrichment was further investigated to confirm the connection between transcriptional regulation and DNA loop structure. As a result of this, we consistently observed that the enrichment of RNA pol II at TSS decreased in SW403 and SW480 cells compared to DLD1 and LS174T cells (Fig. 3C). Taken together, our results demonstrated that CTCF‐mediated DNA loop organization is impacted by p53 activity in CRC cells.

## Discussion

Evolutionary studies have revealed that the *otop2* family (*otop1, 2, 3*) encodes proton‐selective ion channel protein 9, 10. High‐throughput data have demonstrated that *otop2* is highly expressed in colon tissues in TCGA database; however, further research is required to explore implications into phenotype and function. In this study, we found that *otop2* is the most highly down‐regulated gene in CRC (Fig. 1A). Furthermore, we determined that OTOP2 expression is negatively correlated with the malignancy grade and CRC patient survival rates (Fig. 1B,C). This is supported by observations that OTOP2 restrained CRC cell proliferation *in vitro* (Fig. 1D–E). Thus, we suggest that *otop2* is both a novel and an important candidate gene to provide insight into the functional and underlying mechanism in CRC tumorigenesis.

In this study, we discovered that p53 may regulate *otop2* transcription. Initially, we identified varying *otop2* expression levels in DLD1 and SW480 cells with either wtp53 or mtp53 expression (Figs 1D and 2A–C). This inspired us to further study the underlying mechanism of p53‐mediated *otop2* regulation. We confirmed the precise binding sites of p53 and also determined that mtp53 compromised the interaction with the *otop2* promoter. p53‐mediated gene regulation has been well studied in many genes and cancers 11, 12, 13 and is also exhibited by the loss‐of‐function and gain‐of‐function phenomenon to reprogram genomic transcription and drive oncogenesis 14. Here, we suggest that mtp53 loses the ability to activate the transcriptional process of *otop2*, which promotes tumorigenesis.

p53 is a tetramer protein, which binds to specific DNA consensus sequences through cooperative dimer–dimer interaction with a consecutive DNA binding motif as a pair of clamps 15. This induces DNA bending and twisting for spatial re‐organization of regulatory elements 16, where p53 functions as switch to activate and/or silence the initiation of gene transcription. Likewise, CTCF acts as an important insulator to mediate long‐range chromatin looping to form the topological‐associated domain in the genome to systematically control gene transcription 17, 18. However, the mechanism by which p53 drives CTCF to govern DNA loop organization still remains unclear. In this study, we distinguished chromatin of *otop2* promoter interaction between CRC cells expressing either wtp53 or mtp53 (Fig. 3A). Consistent with p53 binding status (Fig. 2E), the promoter interactions of CTCF and RNA pol II were also observed to play a p53‐dependent role in CRC cells (Fig. 3B–C). The p53 CBS of the *otop2* promoter region was observed to be twisted and recruited by wtp53 and CTCF, which altered the physical proximity. Furthermore, wtp53‐induced DNA loop organization provides a suitable environment for *otop2* transcription. Here, we speculate that the lost interaction between mtp53 and the *otop2* promoter programs an aberrant chromatin structure. As a result of this, CTCF fails to form the correct transcriptional environment for RNA pol II recruitment (Fig. 3D). Interestingly, CTCF enrichment patterns mediated by p53 activity (Fig. 3B) and the regulation of chromatin architecture reflect an unexpected diversity across different types of CRC cell lines *in vitro*. Therefore, further investigation is required to determine the implications of other transcription factors, which may also contribute to the DNA loop organization we observed.

Overall, we determined that *otop2* is an important functional candidate in CRC oncogenesis and demonstrated that p53 plays an important role in governing the *otop2* transcriptional process via CTCF binding status reprogramming and the alteration of chromatin architecture.

## Conflict of interest

The authors declare no conflict of interest.

## Author contributions

HQ performed experiments, analyzed the original data, and described the diagrams. YS performed experiments. LY helped PCR experiments. HZ helped manuscript revision. CX designed the overall project and drafted the manuscript.
